# MicroRNA and Rare Human Diseases

**DOI:** 10.3390/genes15101243

**Published:** 2024-09-25

**Authors:** Himanshu Goel, Amy Goel

**Affiliations:** 1 Hunter Genetics, Waratah, NSW 2298, Australia; 2 School of Medicine and Public Health, College of Health, Medicine and Wellbeing, University of Newcastle, Callaghan, NSW 2308, Australia; 3 Billy Blue College of Design, Torrens University Australia, Adelaide, SA 5000, Australia; kurli6@gmail.com

**Keywords:** microRNAs, mRNAs, DICER1 syndrome, DROSHA–DGCR8 complex, RISC, argonaute proteins, gene expression

## Abstract

Background: The role of microRNAs (miRNAs) in the pathogenesis of rare genetic disorders has been gradually discovered. MiRNAs, a class of small non-coding RNAs, regulate gene expression by silencing target messenger RNAs (mRNAs). Their biogenesis involves transcription into primary miRNA (pri-miRNA), processing by the DROSHA–DGCR8 (DiGeorge syndrome critical region 8) complex, exportation to the cytoplasm, and further processing by DICER to generate mature miRNAs. These mature miRNAs are incorporated into the RNA-induced silencing complex (RISC), where they modulate gene expression. Methods/Results: The dysregulation of miRNAs is implicated in various Mendelian disorders and familial diseases, including DICER1 syndrome, neurodevelopmental disorders (NDDs), and conditions linked to mutations in miRNA-binding sites. We summarized a few mechanisms how miRNA processing and regulation abnormalities lead to rare genetic disorders. Examples of such genetic diseases include hearing loss associated with *MIR96* mutations, eye disorders linked to *MIR184* mutations, and skeletal dysplasia involving *MIR140* mutations. Conclusions: Understanding these molecular mechanisms is crucial, as miRNA dysregulation is a key factor in the pathogenesis of these conditions, offering significant potential for the diagnosis and potential therapeutic intervention.

## 1. Introduction

About 20,000–25,000 protein-coding genes make up less than 2% of the human genome [1]. The remaining 98% of the genome was referred to as non-coding DNA. RNA is a highly versatile molecule involved in various cellular functions, including gene regulation and expression. Functional RNAs like mRNA, rRNA, and tRNA were first discovered through their roles in protein synthesis. mRNAs, which encode proteins, are classified as coding RNAs, while non-coding RNAs (ncRNAs) such as rRNA and tRNA have been known for over 60 years for their essential biological roles beyond encoding proteins [2]. Other functional ncRNAs, including imprinted small nucleolar RNAs (snoRNAs), miRNAs, circular RNAs (circRNAs), and long non-coding RNAs (lncRNAs), have been identified and studied. While some, like miRNAs, snoRNAs, and circRNAs, are conserved across species and essential for development, others, like lncRNAs, lack evolutionary conservation. ncRNAs perform various functions, such as modifying rRNAs, regulating mRNA splicing and stability, and modulating chromatin. These activities influence gene expression, impacting cell processes like proliferation, differentiation, apoptosis, and metabolism. Many ncRNAs play regulatory roles in physiological and pathological conditions, contributing to organelle development, stem cell differentiation, aging, cancer, and metabolic disorders. Some have been identified as diagnostic biomarkers and therapeutic targets [3]. The size of RNA molecules varies considerably, depending on their specific function, spanning from a few dozen to several thousand nucleotides. miRNAs are small non-coding RNA molecules, typically comprising approximately 22 nucleotides, that play a critical role in RNA silencing and the post-transcriptional regulation of gene expression [4]. miRNAs regulate gene expression by binding to complementary sequences on target mRNAs, leading to their silencing through mechanisms such as translational repression or mRNA degradation. The biogenesis of miRNAs is a multistep process involving several key enzymes. Recent studies have highlighted the significant impact of miRNA dysregulation on rare, genetically driven disorders, where miRNAs contribute to disease development by disrupting the expression of essential genes [5,6].

## 2. Biogenesis of MiRNA

### 2.1. Transcription and Primary miRNA (pri-miRNA) Formation

miRNA genes are predominantly transcribed by RNA polymerase II, producing primary miRNA (pri-miRNA) transcripts. These pri-miRNAs undergo modifications, including 5′ capping and 3′ polyadenylation. They form stem-loop structures, which are crucial for their subsequent processing.

### 2.2. Nuclear Processing: From pri-miRNA to pre-miRNA

DROSHA is a member of the RNase III family of endonucleases and operates as part of the complex, along with DGCR8. The DROSHA enzyme contains two RNase III domains that cleave double-stranded RNA (dsRNA) molecules, and a dsRNA-binding domain (dsRBD) that helps bind DROSHA to the pri-miRNA.

In the nucleus, the pri-miRNA is processed by a complex composed of DROSHA, an RNase III enzyme, and its cofactor DGCR8 (DROSHA–DGCR8 complex). This complex cleaves the pri-miRNA, releasing a precursor miRNA (pre-miRNA) of approximately 60–70 nucleotides [7]. The cleavage occurs approximately 11 base pairs away from the junction between the single-stranded and double-stranded regions of the RNA. Each of DROSHA’s RNase III domains cuts one strand of the RNA duplex, leaving a 2-nucleotide overhang at the 3′ end of the cleaved product. The pre-miRNA is then transported from the nucleus to the cytoplasm by Exportin-5, a nuclear transport receptor.

### 2.3. Cytoplasmic Processing: From pre-miRNA to Mature miRNA

The pre-miRNA is processed in the cytoplasm by DICER, another RNase III enzyme. DICER cleaves the pre-miRNA, producing an miRNA duplex of about 22 nucleotides with 2-nucleotide overhangs at the 3′ ends [7].

The miRNA duplex is unwound to generate two single-stranded miRNAs. One strand, the guide strand (mature miRNA), is loaded into the RISC, while the other strand, known as the passenger strand (miRNA*), is generally degraded. The mature miRNA can originate from either the 5′ arm or the 3′ arm of the precursor and is therefore labeled as -5p or -3p, respectively. While one arm is integrated into RISC and functions in gene silencing, the other arm is typically a byproduct and is generally degraded. Depending on the tissue or cell type, both the mature miR-5p and miR-3p arms (referred to collectively as the miR-5p/-3p pair) of a pre-miRNA can be associated with RISC.

### 2.4. Incorporation into RISC and Target Regulation

The guide strand is integrated into the RISC, which includes argonaute (AGO) proteins essential for maintaining miRNA stability and facilitating interactions with target mRNAs. The mature miRNA in RISC directs the complex to complementary sequences on target mRNAs, usually within the 3′ untranslated region (3′ UTR). This interaction can result in either mRNA degradation or translational repression, thereby reducing gene expression.

### 2.5. Seed Sequence

**Figure 1:**
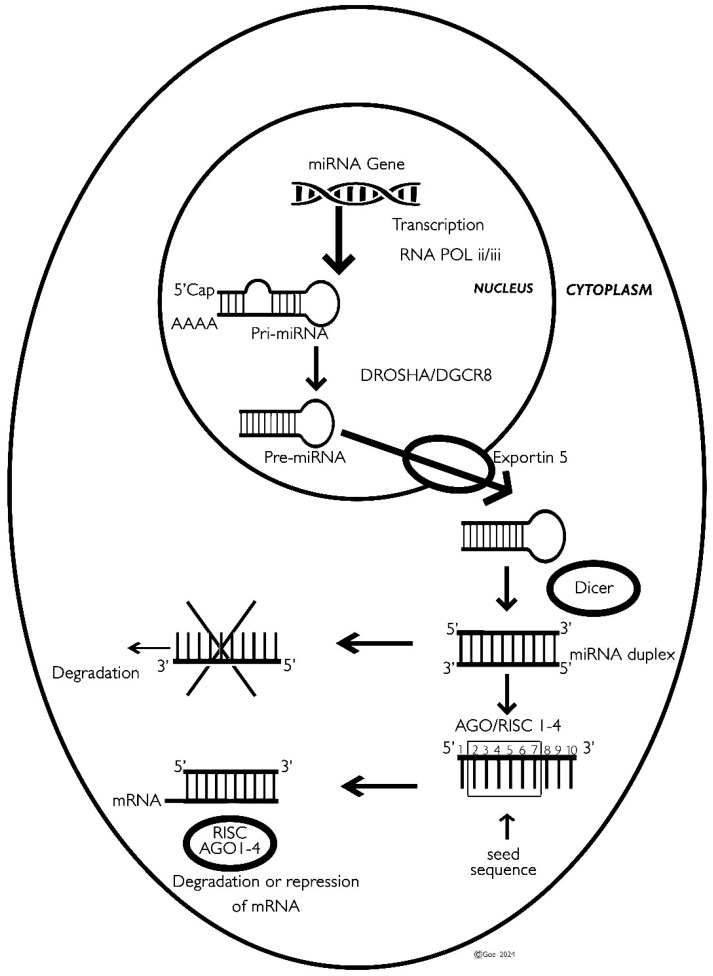
Biogenesis of miRNA: Transcription and primary miRNA formation: miRNA genes are transcribed by RNA polymerase II (or sometimes III) into pri-miRNA transcripts, which are capped and polyadenylated. Nuclear processing: In the nucleus, pri-miRNA is processed by DROSHA and DGCR8 into pre-miRNA, which is then exported to the cytoplasm by Exportin-5. Cytoplasmic processing: In the cytoplasm, DICER processes pre-miRNA into an miRNA duplex. The guide strand (mature miRNA) is loaded into the RNA-induced silencing complex (RISC), while the passenger strand is typically degraded. Incorporation into RISC and target regulation: The guide strand in RISC, containing AGO proteins, guides the complex to complementary sequences on target mRNAs, leading to mRNA degradation or translational repression. Seed sequence: The seed sequence (nucleotides 2–7 from the 5′ end) is crucial for target recognition and miRNA-mediated gene regulation.

The seed sequence, spanning nucleotides 2–7 from the 5′ end of the mature miRNA, is pivotal for target recognition. This sequence pairs with complementary regions on target mRNAs, mainly within the 3′ untranslated region (3′ UTR). The effectiveness and specificity of miRNA-mediated gene regulation are largely determined by the degree of complementarity between the seed sequence and its target sites [8] (Figure 1).

## 3. MicroRNA and Human Diseases

Recently, research has begun to uncover the significant impact of miRNAs on rare disorders, which are diseases that affect a small percentage of the population. These rare disorders often have a genetic basis, and the dysregulation of miRNAs can contribute to their pathogenesis by affecting the expression of key genes [6].

### 3.1. Mendelian Disorders Related to miRNA Biogenesis and Function

The miRNA life cycle involves several key genes, including *DROSHA*, *DGCR8*, *DICER1*, and *AGO1/2* (encoding argonaute protein 1/2). Inherited variants in *DICER1* were found to cause a tumor predisposition disease, commonly referred to as *DICER1* syndrome [6]. In the past two years, research has discovered that germline pathogenic variants (GPVs) in *DGCR8* are associated with tumor predisposition, and that GPVs in *AGO1/2* are linked to neurodevelopmental disorders (NDDs) [9,10].

#### 3.1.1. *DROSHA* and Tumor Predisposition

Somatic mutations in *DROSHA* are frequently found in Wilms’ tumor. These mutations, occurring in the RNase IIIb domain of DROSHA, disrupt metal ion binding and endonuclease activity, leading to the reduced level of several miRNAs, including the tumor suppressor let-7 family. Wilms’ tumor has been associated with a germline *DROSHA* variant, R967W, suggesting that such mutations could potentially predispose individuals to certain tumors [11,12]. Heterozygous null mutations in *DROSHA* have minimal impact on miRNA production. In contrast, heterozygous mutations in the RNase IIIB domain of *DROSHA* exhibit a dominant negative effect, significantly impairing the production of the let-7 family and other miRNAs while still permitting enough residual miRNA processing to support tumor growth. *DROSHA* variants have also been observed in two individuals with severe intellectual disability, epilepsy, white matter atrophy, microcephaly, and dysmorphic features [13].

#### 3.1.2. *DGCR8* and Thyroid Cancer

*DGCR8* is located in the chromosome 22q11.2 region. A recurrent variant in *DGCR8*, c.1552G>A (p.E518K), has been reported in thyroid cancer. This mutation occurs in helix 1 of the first of two double-stranded RNA-binding domains within DGCR8. In silico modeling suggests that the substitution of glutamate (E) with lysine (K) at position 518 likely diminishes RNA binding affinity to DGCR8. The E518K variant results in decreased levels of mature miRNA compared to the normal *DGCR8*, leading to the reduced expression of critical miRNAs in tumors. This reduction is consistent with findings that silencing *DGCR8* promotes tumor growth. Furthermore, the *DGCR8,* E518K mutation has been noted in sporadic cases of follicular thyroid cancer, where an additional loss-of-function (LOF) mutation or loss of heterozygosity (LOH) appears necessary for carcinogenesis [14].

#### 3.1.3. *DICER1* Syndrome

*DICER1* syndrome encompasses a range of rare tumors, including pleuropulmonary blastoma (PPB) and cystic nephroma, as well as the more prevalent euthyroid multinodular goiter (MNG). The GPV for this syndrome is typically a loss-of-function (LOF) variant, most frequently a nonsense or frameshift mutation. For the syndrome to manifest, a second genetic alteration must occur in the *DICER1* gene. This second mutation is often a missense variant that affects crucial residues in ‘hotspots’ within the RNase IIIb domain, which are essential for metal ion binding and endonuclease activity. Although less common, this second hit may also be a loss of heterozygosity (LOH) of the remaining *DICER1* allele. Advances in understanding these genetic events have led to the potential use of circulating tumor DNA (ctDNA) as a tool for screening *DICER1* lesions [15].

#### 3.1.4. Argonaute (AGO) Proteins in NDD

In mammals, four argonaute (AGO) proteins—AGO1, AGO2, AGO3, and AGO4—are fundamental for miRNA-mediated gene regulation. These proteins are essential components of the RISC, where they bind single-stranded miRNAs and guide them to their target mRNAs. Genetic variants in *AGO1* and *AGO2* have been associated with a range of neurodevelopmental disorders (NDDs), including intellectual disability, developmental delays, and speech impairments. These variants disrupt the function of argonaute proteins, impairing their ability to regulate gene expression through miRNA-guided mechanisms.

Lessel et al. identified 13 de novo germline variants in *AGO2* among patients with NDDs, with the majority being missense mutations. Functional assays demonstrated that these variants impair AGO2’s silencing activity to varying extents, mainly through loss-of-function mechanisms. Notably, these variants did not impact AGO2’s slicer activity; instead, they disrupted its ability to efficiently unwind and dissociate from targeted mRNAs after silencing. This resulted in prolonged mRNA association and consequently, impaired gene regulation [9].

Similarly, Schalk et al. reported various GPVs in *AGO1* among NDD probands, emphasizing their impact on neurological development. These *AGO1* variants, including missense mutations and small deletions, were associated with intellectual disabilities and other common clinical features seen in AGO-related disorders [10].

Recent studies suggest that AGO1 and AGO2 may participate in target-directed miRNA decay (TDMD), a mechanism that regulates miRNA turnover and accumulation within cells. The disruption of TDMD by *AGO1* or *AGO2* variants could potentially lead to dysregulated miRNA levels and altered gene expression patterns, contributing to the pathogenesis of NDDs.

Clinically, the identification of *AGO1* and *AGO2* variants expands our understanding of the genetic basis of NDDs, highlighting the importance of genetic testing in patients with unexplained neurodevelopmental or cardiac anomalies. This knowledge enables clinicians to provide more accurate diagnoses, personalized therapeutic strategies, and family counseling based on the specific genetic profiles of affected individuals.

### 3.2. The Role of miRNA Gene Mutations in Mendelian and Inherited Diseases

#### 3.2.1. *MIR96* and Hearing Loss

Nonsyndromic hearing loss (NSHL) is a hereditary condition characterized by hearing impairment without additional symptoms, affecting approximately 1 in 1000 newborns and more than 3 in 1000 adolescents. This condition is genetically heterogeneous, with over 120 identified genes or loci implicated. A specific locus associated with NSHL is DFNA50, located on chromosome 7q32. Initial sequencing efforts within this locus did not reveal causative mutations in protein-coding genes. However, a comprehensive genetic analysis of the miRNA gene *MIR96*, which is also located within this locus, identified two mutations linked to NSHL in two large Spanish families. These mutations exhibited a dominant inheritance pattern [16].

*MIR96* is expressed in cochlear inner and outer hair cells, where it is crucial for their differentiation and plays a role in the development of the auditory hindbrain. Mutations in *MIR96* affect the seed sequence of the mature miRNA (miR-96-5p), which is essential for recognition and binding to target mRNAs.

**Figure 2:**
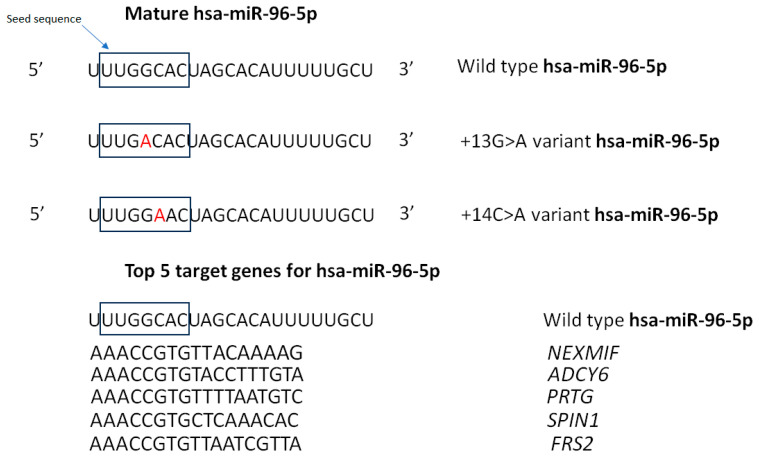
Mature hsa-miR-96-5p and miR-96 (+13G>A) and (+14C>A) mutations are shown to be aligned. The reference base “G” is written in black and the variant base “A” is written in red. The top 5 genes that are regulated by hsa-miR-96-5p genes are also aligned.

Spanish Families: Mutations n.13G>A and n.14C>A in the seed sequence disrupt target recognition. A luciferase reporter assay confirmed the reduced silencing of wild-type miRNA targets by these mutant miRNAs. These mutations also destabilize the miRNA precursor structure, leading to decreased levels of miR-96-5p (Figure 2).

Solda et al. reported a novel variant in *MIR96* that caused autosomal dominant postlingual progressive nonsyndromic hearing loss in an Italian Family. Unlike previous mutations, n.57T>C does not affect the miR-96 seed region or its mature sequence but impairs the maturation of the miR-96 precursor, leading to decreased levels of its mature forms. This mutation is predicted to create a bulge in the miR-96 hairpin structure, potentially disrupting DICER processing.

Mutations in MIR96 are rare causes of deafness, but identifying novel variants like n.57T>C may shed light on the mechanisms underlying DFNA50-associated hearing loss. The n.57T>C mutation appears to cause a quantitative defect in miR-96, leading to a delayed and less severe onset of hearing impairment compared to mutations affecting target recognition. The variability in hearing loss onset and progression among affected families, including incomplete penetrance in some young individuals, suggests that different mutations may contribute to distinct pathogenic mechanisms [17].

Studies in diminuendo mice, which have a similar miR-96-5p seed mutation, showed progressive hearing loss with the upregulation of several wild-type targets, indicating a loss-of-function effect. Additionally, these mice displayed some downregulated genes compared to the wild type, suggesting a possible gain-of-function effect. Mutations in *MIR96* disrupt normal miRNA processing and target regulation, leading to NSHL. These mutations cause structural changes in the miRNA precursor, resulting in decreased levels of mature miRNAs and altered gene regulation. Understanding these mechanisms provides insights into the genetic basis of hearing loss and potential targets for therapeutic intervention [18].

#### 3.2.2. *MIR184* in Eye Diseases

The heterozygous C-to-T transition (n.57C>T) within miR-184 was observed in a large Irish family presenting with autosomal dominant early-onset cataracts and severe keratoconus. The same mutation was also found in a family diagnosed with EDICT syndrome (endothelial dystrophy, iris hypoplasia, congenital cataracts, and stromal thinning) [19]. In both instances, the mutation co-segregated with the disease phenotype, underscoring its pathogenic role in these conditions [20].

The most abundant miRNA in the cornea is miR-184-3p, predominantly situated in the basal and suprabasal layers of the corneal epithelium and within the lens. It is crucial for corneal neovascularization, as well as the proliferation and differentiation of epidermal cells. miR-184-3p competes with miR-205-5p for binding sites, thereby preventing miR-205-5p from downregulating its target genes, *INPPL1* and *ITGB4*, both of which are essential for the proper functioning of corneal basal epithelial hemidesmosomes and the regulation of keratocyte apoptosis.

**Figure 3:**
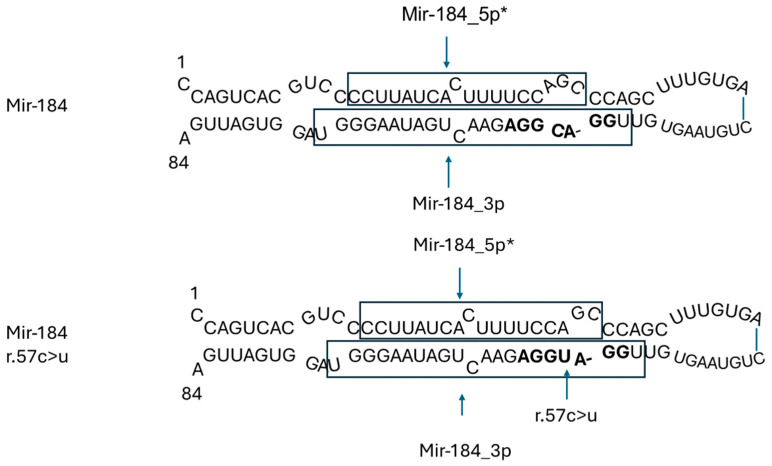
Hughes et al. (2011) identified heterozygosity for a n.57C>T in the *MIR184* precursor sequence. The heterozygous C-to-T transition (n.57C>T) within miR-184 is in the central nucleotide of the functionally essential seven base miRNA seed region (GGACGG) (in bold letters). The mutation was not found in unaffected family members or in controls. Mir-184_5p* is the passenger strand (miRNA*), that is generally degraded.

The heterozygous C-to-T transition (n.57C>T) within miR-184 is in the central nucleotide of the functionally essential seven base miRNA seed region (GGACGG) of mature miR-184-3p. This disrupts the competition with miR-205-5p, thus permitting miR-205-5p to downregulate *INPPL1* and *ITGB4*, which contributes to the pathogenesis of ocular diseases [5,21] (Figure 3).

Two additional mutations in *MIR184* (n.3A>G and n.8C>A) have been identified in families affected by isolated keratoconus [5,22]. Both mutations, miR-184 (n.3A>G) and miR-184 (n.8C>A), disrupt the stem-loop secondary structure of miR-184. The mutation at position 3 alters an adenine residue, which normally pairs with uracil in the wild-type miR-184 stem-loop, to a guanine residue, reducing the base pair probability. Similarly, the mutation at position 8 replaces a cytosine, which typically pairs with guanine in the wild-type structure, with an adenine residue, enlarging the bulge of unpaired residues from six to eight. The structural integrity of miRNA precursors is critical for recognition and cleavage by DROSHA and DICER, proteins essential for miRNA processing. The structural alterations induced by these mutations are likely to interfere with miRNA maturation, potentially affecting miRNA expression and modifying downstream biological processes and pathways [23].

#### 3.2.3. *MIR204* in Inherited Retinal Dystrophy

Inherited retinal dystrophy (IRD) encompasses a diverse group of disorders characterized by the dysfunction or degeneration of photoreceptors, often leading to visual impairment or blindness. A mutation in the miRNA gene has been identified as causative for IRD in a five-generation British family, characterized by an overlapping phenotype of inherited retinal dystrophy, and an optic fissure closure defect, iris coloboma. A large body of evidence indicates that miR-204 plays a crucial role in the differentiation and function of the ocular structures in which it is expressed. In vitro studies have shown that miR-204 may be involved in the correct differentiation and function of RPE cells [24,25,26]. The knockdown of miR-204 function in medaka fish leads to progressive alteration and death of photoreceptor cells [27].

miR-204-5p is highly expressed in the retinal pigment epithelium (RPE) and the ciliary body, with lower expression observed in the inner nuclear layer of the retina and the choroid plexus. It plays a key role in retinal development and function, potentially offering neuroprotection to photoreceptor cells. The n.37C>T mutation does not change the levels of the precursor or mature miR-204-5p, meaning it does not affect the normal production process of the miRNA. However, this lies within the seed region of the 5p arm; this change would affect the recognition of target genes. This could occur through two different modalities: either the impaired recognition of bona fide miR-204 targets (loss-of-function mechanism) or the creation of novel aberrant target sites (gain-of-function mechanism) [28]. miR-204 is located within intron 8 of the TRP channel gene, *TRPM3*, on chromosome 9q21.12. Deletions within 9q21 and those specifically encompassing the *TRPM3* gene have not been reported to cause ocular phenotypes, instead causing features of mental retardation, epilepsy, speech delay, autistic behavior, and moderate facial dysmorphism [29,30,31,32]. The mutation (n.37C>T) occurs in the 4th position of the miR-204-5p seed sequence. It exhibits autosomal dominant inheritance within the affected family members, perfectly segregating with the disease phenotype [33]. This indicates that the n.37C>T mutation acts via a gain-of-function mechanism.

Further research is needed to identify specific targets of the mutant miRNA and explore potential therapeutic strategies targeting miR-204-5p regulation in IRD.

#### 3.2.4. *MIR140* in Skeletal Dysplasia

The skeletal system is composed of bones, cartilage, joints, bone marrow, tendons, and ligaments. Disruptions in gene expression during its development can result in defective bone formation, cartilage abnormalities, skeletal mis-patterning, and joint malformations. Skeletal dysplasia encompasses a highly heterogeneous group of genetic disorders, affecting cartilage and bone development. These disorders are caused by mutations in various genes, including in the miRNA gene *MIR140*. miR-140 is one of the most extensively studied miRNAs that are associated with cartilage development. miR-140 is believed to be expressed explicitly in cartilage but not in other tissues based on whole-mount in situ hybridization data in zebrafish and mouse embryos [34,35]. miR-140-3p is the predominant microRNA in cartilage, with levels about ten-fold higher than miR-140-5p [36]. Both strands, miR-140-3p and miR-140-5p, play functional roles in regulating cartilage development and homeostasis. Studies in knockout mice suggest that miR-140-3p is crucial for maintaining proper cartilage function, and its absence leads to a mild skeletal phenotype [36].

A novel skeletal dysplasia was discovered in two unrelated families through a molecular diagnostic project focused on ultra-rare congenital skeletal disorders. The clinical phenotype included disproportionate short stature, shortened limbs, small hands and feet, and midface hypoplasia with a notably small nose. Radiological findings revealed mild spondylar dysplasia, delayed epiphyseal ossification in the hip and knee joints, and severe brachydactyly with cone-shaped phalangeal epiphyses. These skeletal abnormalities progressed to premature spondylosis and degenerative joint disease in adulthood. All affected individuals had normal cognitive function, dentition, hearing, and visual acuity. Whole-genome sequencing identified a shared heterozygous nucleotide substitution (chr16:69967007A>G, *MIR140*: n.24A>G) in both families. This variant affects the first nucleotide of the seed sequence of the highly conserved miRNA, miR-140-5p, encoded by *MIR140* [37].

This mutation underscores the intricate role of miRNAs in skeletal development and homeostasis. A detailed understanding of the specific targets and molecular pathways disrupted by such mutations is essential for developing targeted therapies for skeletal dysplasia disorders [36,37].

#### 3.2.5. *MIR17HG*: Feingold Syndrome Type 2

Feingold syndrome is an autosomal dominant genetic disorder characterized by learning disabilities, short stature, microcephaly, and brachymesophalangy. Pathogenic variants in the *MYCN* gene are the main cause of Feingold syndrome; some cases without *MYCN* mutations have been linked to microdeletions in the 13q31-q32 region, particularly affecting *MIR17HG*, which encodes the miR-17–92 cluster [38].

Whole-genome comparative genome hybridization (CGH) analysis revealed microdeletions ranging from 165 kb to 17 Mb in the 13q31-q32 region in patients with skeletal abnormalities resembling Feingold syndrome, who did not have *MYCN* mutations. These microdeletions were found to segregate with the disease phenotype within affected families, implicating the haploinsufficiency of *MIR17HG* as the underlying cause. The minimal overlapping region of these deletions included *MIR17HG*, leading to the classification of these cases as Feingold syndrome type 2 (FGLDS2). *MIR17HG* encodes the miR-17–92 cluster, also known as OncomiR-1 due to its oncogenic roles in various cancers. In normal cells, this miRNA cluster is crucial for the development of the lung, heart, skeleton, and immune system. The loss of one copy of *MIR17HG* due to these microdeletions results in haploinsufficiency, which disrupts the normal developmental processes regulated by the miR-17–92 cluster [39,40].

Conversely, microduplications in the 13q31 region overlapping *MIR17HG* have been observed in patients with contrasting phenotypes. These patients exhibit a tall stature, macrocephaly, developmental delay, and skeletal abnormalities, indicating that the overexpression of the miR-17–92 cluster due to microduplications can lead to distinct clinical manifestations [41].

#### 3.2.6. Copy Number Variations in *MIR9-3* and *MIR1299* Congenital Anomalies of the Kidney and Urinary Tract (CAKUTs)

CAKUTs are structural and functional abnormalities affecting about 1 in 500 liveborn children and are the leading cause of pediatric kidney failure. Genetic factors, including point mutations and rare copy number variants (rCNVs), account for 20–25% of CAKUT cases. Notably, miRNA genes are enriched in rCNVs and common copy number variations (cCNVs) associated with CAKUTs. miRNAs in these regions regulate key cellular processes such as growth, differentiation, inflammation, and apoptosis, which are crucial for CAKUT development. Copy number variation involving *MIR9-3* and *MIR1299* showed significant association with CAKUTs. Although miRNAs have been proposed as potential genetic factors in genotype–phenotype studies, their specific role in CAKUTs remains unclear [42].

### 3.3. Human Diseases Associated with Variants in miRNA-Binding Sites

Given that mutations in the seed regions of *miRNA* genes can cause human genetic diseases, it can be expected that the variants in the miRNA-binding sites in the 3′UTRs of mRNAs may also be associated with human diseases. miRNAs regulate gene expression by binding to the mRNA. The seed sequence is essential for the binding of the miRNA to the mRNA. The seed sequence or seed region is a conserved heptameric sequence, which is mostly situated at positions 2–7 from the miRNA 5′-end. Even though the base pairing of miRNA and its target mRNA does not match perfectly, the “seed sequence” must be perfectly complementary.

Variants in miRNA-binding sites within the 3′ untranslated regions (3′UTRs) of mRNAs can significantly contribute to human diseases by altering the normal regulation of gene expression. Here is a comprehensive explanation based on three representative examples:

#### 3.3.1. The Variants in miR-189-Binding Site of *SLITRK1* Gene Associated with Tourette’s Syndrome (TS)

Tourette’s syndrome (TS) is a genetically influenced developmental neuropsychiatric disorder characterized by chronic vocal and motor tics. The Slit and Trk-like 1 (*SLITRK1*) gene on chromosome 13q31.1 has been assessed as a candidate gene due to its proximity to a de novo chromosomal inversion observed in a child with TS. Among 174 unrelated probands, researchers identified a single-base deletion in the coding region leading to a frameshift, predicted to result in a truncated protein [43]. In addition to this frameshift mutation, another non-coding sequence variant was identified in two unrelated individuals with TS. The single-base change was present in the 3′ untranslated region (UTR) of the transcript and corresponds to a highly conserved nucleotide within the predicted binding site for the human miRNA hsa-miR-189. This variant replaces a G:U wobble base pair with an A:U Watson–Crick pairing at position 9 in the miRNA-binding domain. G:U wobble base pairs inhibit miRNA-mediated protein repression, suggesting that this variant might affect *SLITRK1* expression [43].

*SLITRK1* expresses significantly in brain regions previously implicated in TS and it has an important role in neurite outgrowth [43].

#### 3.3.2. Variants in miR-196-Binding Site of *IRGM* Gene Associated with Crohn’s Disease (CD)

Crohn’s disease (CD), a form of inflammatory bowel disease (IBD), has been linked to a polymorphism in the coding region of the *IRGM* gene. This synonymous exonic variant, CTG (Leucine)/TTG (Leucine), occurs at the miR-196-binding site, altering miRNA-mediated regulation. The c.313C>T polymorphism of *IRGM* is located within the seed region, where an mRNA–miRNA complex forms within RISC, which is important for mRNA regulation. The variant decreases miR-196-binding efficiency, resulting in elevated *IRGM* expression in inflamed intestinal epithelial cells of CD patients. This dysregulation impairs autophagy, a crucial immune mechanism for pathogen defense. The increased IRGM expression due to this polymorphism may play a role in the pathogenesis of CD by compromising immune homeostasis, thereby contributing to the disease’s onset and progression. Recently, miR-29 was found to have a role in Crohn’s disease through its regulation of fibrosis and inflammation. miR-29 is known for its ability to suppress the expression of pro-fibrotic genes such as collagen and transforming growth factor-β (TGF-β) [44].

#### 3.3.3. Mutations in miR-433-Binding Site of *HDAC6* Gene Associated with X-Linked Dominant Chondrodysplasia

**Figure 4:**
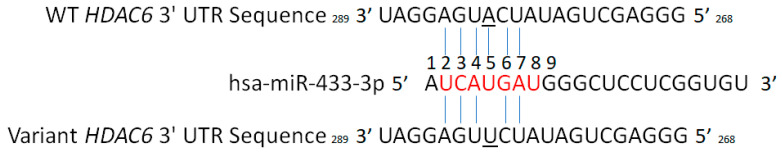
A variant in the 3′-UTR of the *HDAC6* gene in a proband with X-linked dominant chondrodysplasia is depicted here. The sequence alignment of the hsa-miR-433-3p with the wild-type (WT) and variant *HDAC6* 3′ UTR region of mRNA are shown here concerning the seed region of miRNA that is shown in red. The *HDAC6* variant is within the 3′-UTR A>T mutation. The variant has loosened the binding of miRNA with the *HDAC6* 3′ UTR mRNA. Blue vertical lines denote the hydrogen bonds between *HDAC6* DNA and miR-433 RNA seed sequence. Underlined bases represent the sequence change in *HDAC6* (3′-UTR A>T mutation).

The genes implicated in chondrodysplasias are involved in encoding extracellular matrix proteins, signaling molecules, transcription factors, or components of metabolic pathways. A family with X-linked dominant chondrodysplasia was found to have a novel variant, c.*281A>T in the 3′ untranslated region (UTR) of the *HDAC6* gene that segregates well with the disease in the family. This variant lies within the binding site, or seed sequence, of miR-433, responsible for the post-transcriptional regulation of *HDAC6* expression. The mutation disrupts the miR-433-mediated post-transcriptional regulation of *HDAC6*, resulting in elevated levels of *HDAC6* expression in affected individuals. This dysregulation of *HDAC6* has profound effects on cartilage and bone development, as *HDAC6* influences processes such as cell differentiation, cytoskeletal organization, and protein turnover. The c.*281A>T variant lies within a sequence matching the seed of miR-433. The predicted miR-433 site in the *HDAC6* 3′UTR contains the miRNA seed sequence (miRNA nucleotides 2–7; 5′UCAUGAUG3′). A perfect and contiguous match to miRNA nucleotides 2–7 is crucial for the biological and functional properties of miRNAs, and any mismatch in that sequence greatly affects gene expression regulation. The c.*281A>T variant changes the predicted miRNA site from 5′AUCAUGA3′ to 5′AUCUUGA3′, disrupting miR-433 binding and leading to increased *HDAC6* expression [8,45] (Figure 4).

We have summarized the known miRNA-related genetic disorders in Table 1.

With advances in sequencing technology, many researchers have developed databases to store information on miRNAs and diseases. These databases have greatly benefited computational biology by providing valuable data. Key databases include: [46]

HMDD: Human MicroRNA Disease Database (http://www.cuilab.cn/hmdd, accessed on 19 August 2023) [47];

dbDEMC: Database of Differentially Expressed miRNAs in Cancers (https://www.biosino.org/dbDEMC, accessed on 19 August 2023) [48];

miR2Disease: miRNA and Disease Database (http://www.mir2disease.org/) [49];

miRCancer: miRNA and Cancer Database (http://mircancer.ecu.edu/) [50].

These resources are frequently used for predicting miRNA-disease associations (MDAs).

## 4. Conclusions

Pathogenic variants in genes involved in miRNA biogenesis, processing, and gene expression process are not well reported in the literature. miRNAs play a crucial role in regulating gene expression and have been increasingly recognized as key contributors to various genetic diseases. Their ability to target multiple mRNAs and influence a wide range of cellular processes highlights their complexity and importance in both normal physiology and disease pathology. Mutations in miRNA genes and their associated regulatory pathways can lead to the dysregulation of critical genes, contributing to the development of conditions such as neurodevelopmental disorders and inherited syndromes. The emerging evidence linking miRNAs to genetic diseases underscores the importance of identifying miRNA–mRNA interactions in understanding the molecular basis of these disorders. Advances in sequencing technologies and bioinformatics tools have made it possible to investigate the full spectrum of miRNA targets, providing valuable insights into the pathological mechanisms involved. However, despite the progress in identifying disease-associated miRNAs, many challenges remain in fully elucidating their roles in disease pathogenesis.

Overall, miRNA research holds tremendous potential for advancing our knowledge of genetic diseases and improving patient outcomes through innovative diagnostic and therapeutic approaches.

## Figures and Tables

**Table 1:** Table summarizing the genes related to miRNAs and their associated rare diseases.

miRNA Gene	Associated Disease	Mutation Type	Mechanism
*DROSHA*	Wilms’ tumor	Nonsense/missense	Defective miRNA processing
*DGCR8*	Thyroid tumor	Missense and second hit mutation	Decreased levels of mature miRNA
*DICER1*	Rare tumors like pleuropulmonary blastoma	Germline nonsense/frameshift leading to loss of function with second hit mutation	Decrease in mature miRNA
*AGO1*	NDDs	Missense and small deletions	Defecting RISC processing
*AGO2*	NDDs	Missense variants	Defecting RISC processing
*MIR96*	Nonsyndromic hearing loss (NSHL)	Point mutations (n.13G>A, n.14C>A)	Disrupts miRNA seed sequence, affecting mRNA regulation
*MIR184*	Keratoconus and cataracts	n.57C>T	Alters seed sequence, disrupts competition with miR-205-5p for target mRNAs
*MIR204*	Inherited retinal dystrophy, iris coloboma	n.37C>T	Gain-of-function mutation alters recognition of target genes
*MIR140*	Skeletal dysplasia	n.24A>G	Seed sequence mutation affects cartilage development
*MIR17HG*	Feingold syndrome type 2	Deletion or microduplication	Alters miR-17–92 cluster expression, impacting developmental processes
*MIR9-3, MIR1299*	Congenital Anomalies of Kidney and Urinary Tract (CAKUTs)	Copy number variations	Affects kidney development through altered miRNA-mediated gene regulation
*SLITRK1 (miR-189-binding site)*	Tourette’s syndrome	Single-base variant in 3′UTR	Disrupts miRNA binding, altering SLITRK1 expression
*IRGM (miR-196-binding site)*	Crohn’s disease	Synonymous variant (c.313C>T)	Reduces miR-196 binding, increasing IRGM expression, affecting autophagy
*HDAC6 (miR-433-binding site)*	X-linked chondrodysplasia	c.*281A>T in 3′UTR	Disrupts miR-433 binding, leading to overexpression of HDAC6

Nucleotides downstream (3′) of the translation termination codon (stop) are marked with a * (asterisk) and numbered c.*1, c.*2, c.*3, etc., going further downstream.

## Data Availability

No new data were created or analyzed in this study. Data sharing is not applicable to this article.

## Abbreviations

List of Abbreviations Used in this Article.

**Abbreviation**	**Definition**
miRNAs	microRNAs
pri-miRNA	primary miRNA
DGCR8	DiGeorge syndrome critical region 8
RISC	RNA-induced silencing complex
NDDs	neurodevelopmental disorders
ncRNAs	non-coding RNAs
mRNAs	messenger RNAs
snoRNAs	small nucleolar RNAs
circRNAs	circular RNAs
lncRNAs	long non-coding RNAs
dsRBD	dsRNA-binding domain
dsRNA	double-stranded RNA
pre-miRNA	precursor miRNA
AGO	argonaute AGO
3′ UTR	3′ untranslated region
EDICT syndrome	endothelial dystrophy, iris hypoplasia, congenital cataracts, and stromal thinning
